# *Hmga2* is required for canonical WNT signaling during lung development

**DOI:** 10.1186/1741-7007-12-21

**Published:** 2014-03-24

**Authors:** Indrabahadur Singh, Aditi Mehta, Adriana Contreras, Thomas Boettger, Gianni Carraro, Matthew Wheeler, Hector A Cabrera-Fuentes, Saverio Bellusci, Werner Seeger, Thomas Braun, Guillermo Barreto

**Affiliations:** 1 LOEWE Research Group Lung Cancer Epigenetic, Max-Planck-Institute for Heart and Lung Research, Parkstraße 1, 61231 Bad Nauheim Germany; 2 Department of Cardiac Development and Remodeling, Max-Planck-Institute for Heart and Lung Research, Parkstraße 1, 61231 Bad Nauheim, Germany; 3 Chair for Lung Matrix Remodeling, Excellence Cluster Cardio Pulmonary System, Justus-Liebig- University, 35932 Giessen, Germany; 4 Biochemistry Institute, Medical School, Justus-Liebig- University, 35932 Giessen, Germany; 5 Institute of Fundamental Medicine and Biology, Kazan (Volga Region) Federal University, 18 Kremlyovskaya Street, Kazan 420008, Russian Federation; 6 Department of Lung Development and Remodeling, Max-Planck-Institute for Heart and Lung Research, Parkstraße 1, 61231 Bad Nauheim, Germany

**Keywords:** Branching morphogenesis, HMGA2, GATA6, Lung development, WNT signaling

## Abstract

**Background:**

The high-mobility-group (HMG) proteins are the most abundant non-histone chromatin-associated proteins. HMG proteins are present at high levels in various undifferentiated tissues during embryonic development and their levels are strongly reduced in the corresponding adult tissues, where they have been implicated in maintaining and activating stem/progenitor cells. Here we deciphered the role of the high-mobility-group AT-hook protein 2 (HMGA2) during lung development by analyzing the lung of *Hmga2*-deficient mice (*Hmga2*^*−/−*^).

**Results:**

We found that *Hmga2* is expressed in the mouse embryonic lung at the distal airways. Analysis of *Hmga2*^*−/−*^ mice showed that *Hmga2* is required for proper cell proliferation and distal epithelium differentiation during embryonic lung development. *Hmga2* knockout led to enhanced canonical WNT signaling due to an increased expression of secreted WNT glycoproteins *Wnt2b*, *Wnt7b* and *Wnt11* as well as a reduction of the WNT signaling antagonizing proteins GATA-binding protein 6 and frizzled homolog 2. Analysis of siRNA-mediated loss-of-function experiments in embryonic lung explant culture confirmed the role of *Hmga2* as a key regulator of distal lung epithelium differentiation and supported the causal involvement of enhanced canonical WNT signaling in mediating the effect of *Hmga2*-loss-of-fuction. Finally, we found that HMGA2 directly regulates *Gata6* and thereby modulates *Fzd2* expression.

**Conclusions:**

Our results support that *Hmga2* regulates canonical WNT signaling at different points of the pathway. Increased expression of the secreted WNT glycoproteins might explain a paracrine effect by which *Hmga2*-knockout enhanced cell proliferation in the mesenchyme of the developing lung. In addition, HMGA2-mediated direct regulation of *Gata6* is crucial for fine-tuning the activity of WNT signaling in the airway epithelium. Our results are the starting point for future studies investigating the relevance of *Hmga2*-mediated regulation of WNT signaling in the adult lung within the context of proper balance between differentiation and self-renewal of lung stem/progenitor cells during lung regeneration in both homeostatic turnover and repair after injury.

## Background

The mouse lung arises from the anterior endoderm and forms during five overlapping phases of lung development: embryonic (embryonic days post coitum (E) 9 to 12.5), pseudoglandular (E12.5 to E16.5), canalicular (E16.5 to E17.5), saccular (E17.5 to post-natal day (P) 5) and alveolar (P5 to P28) [1-3]. At the end of the embryonic phase, primary and secondary lung buds formation has taken place and the embryonic lung consists of one left lobe and four right lobes. From E10.5 to E16.5, the epithelium undergoes branching morphogenesis to form the respiratory (bronchial) tree. In parallel to branching morphogenesis, the airway epithelium differentiates from a morphologically uniform cell population to different specialized cell types, thereby establishing a proximal-distal axis in the developing lung. However, most of the differentiation occurs in the canalicular and saccular phases (E16.5 to P5). The primitive lung epithelium co-expresses several lineage markers including Clara cell-specific 10 kDa protein (*Scgb1a1*, also CC10) and surfactant-associated protein C (*Sftpc*, also SP-C). Later in gestation (E16.5 onwards), *Scgb1a1* is a marker for the proximal epithelium, whereas *Sftpc* expression defines the distal epithelium. In the adult lung these markers are characteristic of distinct cell lineages, *Scgb1a1* of Clara cells and *Sftpc* of alveolar type II cells. Only specific progenitor cells in the adult lung, bronchioalveolar stem cells (BASCs), co-express *Scgb1a1* and *Sftpc*[4].

Several evolutionarily conserved signaling pathways have been implicated in different phases of embryonic lung development. In particular, members of the fibroblast growth factor, bone morphogenetic protein, hedgehog/Gli, epidermal growth factor and wingless secreted glycoprotein (WNT) families have been implicated in lung morphogenesis and epithelial differentiation [2,5-7]. In addition, a well-organized and balanced interplay between these signaling pathways and key transcription factors of lung development, including NK2 homeobox 1 (also known as thyroid transcription factor 1), forkhead box protein A2 (also known as hepatocyte nuclear factor 3-beta) and GATA6, is required for proper lung formation [2,3,7]. GATA6 is the only member of the GATA family of zinc finger transcription factors that is expressed in the distal epithelium of the developing lung [8,9]. GATA6 is essential for branching morphogenesis and regulates differentiation of distal lung epithelium [9,10]. Moreover, GATA6 has been implicated in blocking WNT signaling to control the balance between BASC expansion and lung epithelial differentiation required for both lung development and regeneration [11].

High mobility group AT-hook protein 2 (HMGA2) is a transcription regulator belonging to the family of HMG proteins. HMG proteins are the most abundant non-histone chromatin-associated proteins and regulate gene expression by altering chromatin structure and recruiting other proteins to the transcription regulatory complex [12]. HMGA2 is present at high levels in various undifferentiated tissues during embryonic development and its levels are strongly reduced in the corresponding adult tissues [12,13]. In addition, *Hmga2* expression in adult organs has been implicated in maintaining and activating stem/progenitor cells in different tissues [14,15]. Here, we show that *Hmga2* mRNA levels are high during early stages of lung development, in which cells are undifferentiated, and become reduced and restricted to the distal airways as lung development progresses, coincident with cell differentiation. Analysis of the lung of *Hmga2*-knockout (KO) mice [16] revealed enhanced canonical WNT signaling that led to increased cell proliferation, increased number of progenitor cells and reduced differentiation of the distal airway epithelium. Using a lung explants culture system, we confirmed the causal involvement of WNT signaling mediating the effect of *Hmga2*-loss-of-function (LOF) and showed that *Hmga2* is required for proper branching morphogenesis during the formation of the bronchial tree. Furthermore, we showed that *Hmga2* regulates canonical WNT signaling at different points of the pathway. Increased expression of the secreted WNT glycoproteins might explain a paracrine effect by which *Hmga2*-KO enhanced cell proliferation in the mesenchyme of the developing lung. In addition, HMGA2-mediated direct regulation of *Gata6* is crucial for fine-tuning the activity of WNT signaling in the airway epithelium.

## Results

### *Hmga2* is expressed in the mouse embryonic lung at the distal airways

**Figure 1:**
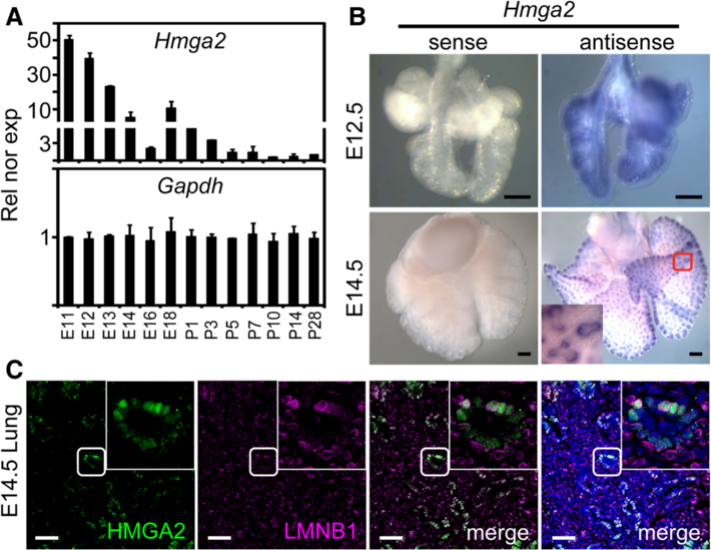
***Hmga2 *is expressed in the mouse embryonic lung at the distal airways. (A)***Hmga2* is expressed during embryonic lung development. *Hmga2* and *Gapdh* expression was monitored by quantitative RT-PCR in mouse embryonic lung at different days post coitum (E11.5 to E18.5) and in mouse lung at different days after birth (P1 to P28). Rel nor exp, relative expression normalized to *Tuba1a*. Error bars show the SEM (*n* = 4). **(B)***Hmga2* is expressed in the mouse embryonic lung at the distal airways. *Hmga2* mRNA was detected in mouse embryonic lung at E12.5 and E14.5 by *in situ* hybridization using an *Hmga2*-specific antisense RNA probe. Sense probe, negative control. Scale bars, 200 μm. Square shows details at higher magnification. **(C)** HMGA2 localized in the cell nuclei of the embryonic lung. Fluorescence microscopy of embryonic lung sections (E14.5) after double immunostaining using HMGA2- and LMNB1-specific antibodies. Nuclear staining with DAPI (blue). Squares as in B. Scale bars, 40 μm.

To verify that *Hmga2* is expressed during lung development, quantitative reverse transcription PCR (qRT-PCR) expression analysis was performed (Figure 1A). *Hmga2* transcript was detected at E11.5, when the primary lung buds have evaginated from the foregut and secondary buds are forming as outgrowths from the primary lung buds. *Hmga2* expression progressively decreased during the pseudoglandular stages of lung development (E12.5 to E16.5). Between the canalicular (E16.5 to E17.5) and initial saccular stages (E17.5 to E18.5), the levels of *Hmga2* transcript increased again. Later in gestation (saccular stages, E18.5 to P5), *Hmga2* expression was further reduced and reached a basal level of expression that was maintained through the alveolar phase (P5 to P28). Thus, *Hmga2* mRNA levels were high during early stages of lung development, in which cells are undifferentiated, and decreased as lung development progressed, coincident with cell differentiation.

*In situ* hybridization expression pattern analysis in the embryonic lung at E12.5 (Figure 1B), when branching morphogenesis of the lung bud is proceeding rapidly to establish the future bronchial tree, revealed that *Hmga2* is ubiquitously expressed with higher levels of expression at the tips of the growing lung buds. Interestingly, *Hmga2* expression became restricted to the distal lung endoderm at E14.5. Consistently, immunostaining on sections of the embryonic lung at E14.5 (Figure 1C) supported the presence of HMGA2 in cells of the distal lung endoderm. Co-staining with an antibody specific for the nuclear envelope protein lamin B1 (LMNB1) demonstrated the nuclear localization of HMGA2. The observed expression patterns in embryonic lung suggest a role for HMGA2 in epithelial differentiation.

### *Hmga2* is required for proper differentiation of the distal epithelium during lung development

**Figure 2:**
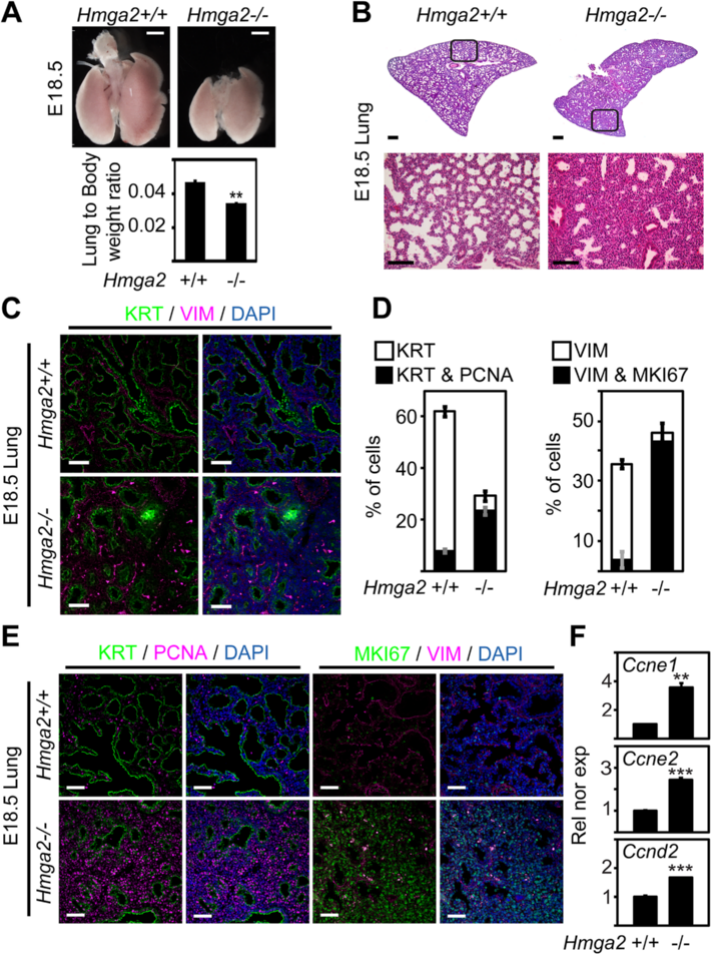
***Hmga2***^***−/−***^**mice embryonic lung showed increased cell proliferation. (A)** Lungs of *Hmga2*-KO (−/−) embryo were smaller (top) and showed a reduced lung-to-body-wet-weight ratio (bottom) when compared to WT (+/+) embryos at E18.5. Scale bars, 1 mm. Error bars show the SEM (*n* = 6). ***P <0.001; **P <0.01; *P <0.05 after one-way analysis of variance. **(B)** Representative of histological analysis using hematoxylin and eosin stain on sections of WT and *Hmga2*-KO embryonic lung at E18.5. Squares are shown at the bottom at higher magnification. Scale bars, 100 μm. **(C)** Sections of embryonic lung (E18.5) of WT and *Hmga2*-KO mice were analyzed by confocal microscopy after double immunostaining using KRT- and VIM-specific antibodies. Nuclear staining with DAPI (blue). Scale bars, 40 μm. **(D)** Quantification of proliferating (PCNA- or MKI67-positive) epithelial (KRT) and mesenchymal (VIM) cells showed increased proliferation in both tissues of *Hmga2-*KO embryonic lung. Sections of embryonic lung (E18.5) of WT and *Hmga2*-KO mice were treated as in E and used for quantification. Error bars show the SEM (*n* = 3). **(E)** Sections of embryonic lung (E18.5) of WT and *Hmga2*-KO mice were analyzed as in C using (left) KRT- and PCNA-specific antibodies or (right) VIM- and MKI67-specific antibodies. Nuclear staining with DAPI (blue). Scale bars, 40 μm. **(F)***Hmga2*-KO enhanced expression of cell-cycle progression markers. Expression analysis of the indicated genes by quantitative RT-PCR in E18.5 lung of WT and *Hmga2*-KO mice. Rel nor exp, relative expression normalized to *Tuba1a*. Error bars show the SEM (*n* = 4). Asterisks as in A.

To determine the role of *Hmga2* during lung development, we analyzed the embryonic lung of *Hmga2*-deficient mice (*Hmga2*^*−/−*^) [16]. At E18.5, when the bronchial tree is complete and the lung tissues are differentiating into different cell types that will constitute the lung after birth, *Hmga2*-KO resulted in a reduced body weight and lung-to-body-wet-weight ratio (Additional file 1: Figures S1A and Figure 2A). Macroscopically, the embryonic lung of *Hmga2*-KO mice at E18.5 had four lobes on the right side and one lobe on the left, indicating that the earliest events during lung development, specification of pulmonary endoderm and primary branching morphogenesis, occurred normally and do not require *Hmga2*. However, histological analysis of the embryonic lung at this stage (Figure 2B) revealed a marked increase of cell density in the *Hmga2*^*−/−*^ mice when compared with the wild-type (WT) mice, suggesting increased cell proliferation. Furthermore, immunostaining on sections of embryonic lung (Figure 2C) using antibodies specific for the epithelial marker pan-cytokeratin (KRT) and the mesenchymal marker vimentin (VIM) showed a broader mesenchyme and an irregularly shaped epithelium in *Hmga2*-KO mice when compared to the WT mice. This suggests an expansion of the mesenchyme at the expense of the epithelium. Consistent with this observation, quantification of KRT-positive cells (Figure 2D, left) showed a decrease from 61.7% to 29.5% (*P* <0.001; *n* = 3), whereas the number of VIM-positive cells (right) increased from 35.7% to 46.3% (*P* <0.001; *n* = 3) in *Hmga2*-KO mice when compared to the WT mice. Immunostaining on sections of embryonic lung showed increased levels of proliferation markers, specifically proliferating cell nuclear antigen (PCNA) and antigen identified by monoclonal antibody Ki 67 (MKI67) (Figure 2E and Additional file 1: Figure S1C), in both the epithelium and the mesenchyme of the *Hmga2*-KO mice, as shown by co-staining with antibodies specific for KRT (left) or VIM (right), supporting our interpretation of the histological analysis. However, quantification of our results (Figure 2D) showed more prominent cell proliferation in the mesenchyme (right), from 3.9% to 43.3% (*P* <0.001; *n* = 3) when compared to the epithelium (left), from 8% to 23.6% (*P* <0.001; *n* = 3). To confirm increased cell proliferation in the embryonic lung after *Hmga2*-KO, we analyzed the expression of cell-cycle progression markers cyclin E1, E2 and D2 (Figure 2F). Expression of these genes increased after *Hmga2*-KO when compared to WT mice, demonstrating elevated cell proliferation.

**Figure 3:**
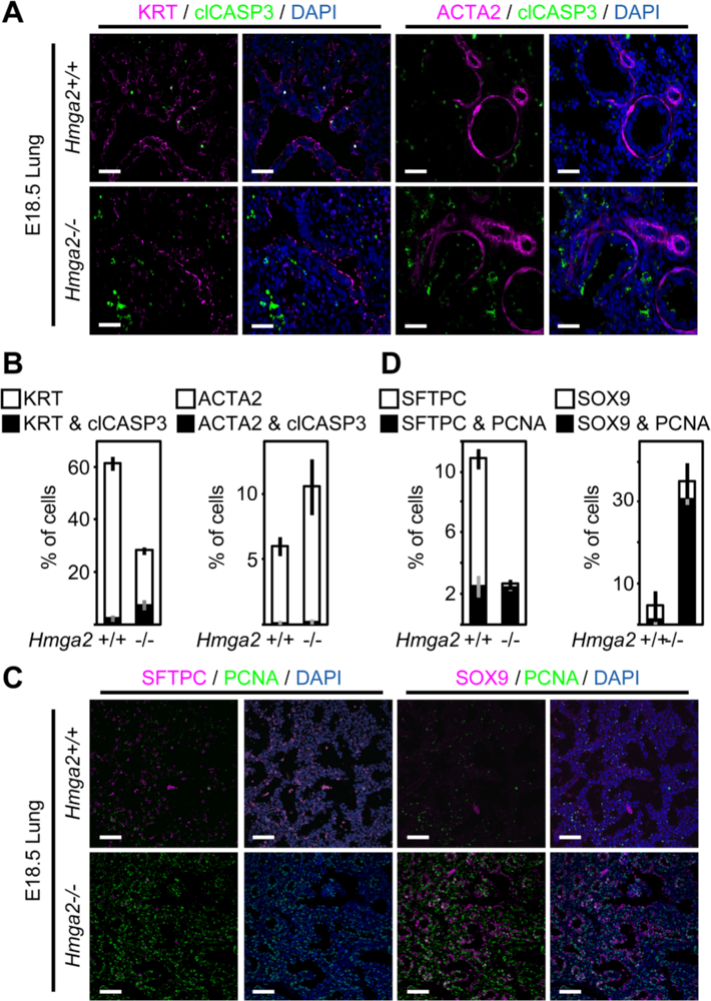
***Hmga2 *is required for proper differentiation of the distal epithelium. (A)** Sections of embryonic lung (E18.5) of WT and *Hmga2-*KO mice were analyzed as in Figure 2C using clCASP3- and KRT- (left) or ACTA2- (right) specific antibodies. Nuclear staining with DAPI (blue). Scale bars, 40 μm. **(B)** Quantification of clCASP3 positive epithelial (KRT) and mesenchymal (ACTA2) cells showed increased apoptosis in epithelial cells of *Hmga2-*KO mice embryonic lung. Sections of embryonic lung (E18.5) of WT and *Hmga2*-KO mice were treated as in A and used for quantification. Error bars show the SEM (*n* = 3). **(C)** Sections of embryonic lung (E18.5) of WT and *Hmga2-*KO mice were analyzed as in Figure 2C using (left) SFTPC- and PCNA-specific antibodies or (right) SOX9- and PCNA-specific antibodies. Nuclear staining with DAPI (blue). Scale bars, 40 μm. **(D)** Quantification of proliferating SFTPC- and SOX9-positive cells showed reduced differentiation and increased proliferation of progenitor cells in the distal epithelium of *Hmga2-*KO mice embryonic lung. Sections of embryonic lung (E18.5) of WT and *Hmga2*-KO mice were treated as in C and used for quantification. Error bars show the SEM (*n* = 3).

In addition to cell proliferation, programmed cell death is an important process involved in lung development [17]. Thus, we performed co-staining on sections of embryonic lung (Figure 3A) using antibodies specific for the active form of the apoptosis-related cysteine peptidase, cleaved caspase 3 (clCASP3) and KRT (left) or the mesenchymal marker smooth muscle actin alpha 2 (ACTA2, right). Quantification of clCASP3-positive cells (Figure 3B) showed that whereas the level of apoptosis in the mesenchyme was not significantly affected after *Hmga2*-KO (0.2% in the WT versus 0.3% in the KO), it increased in the lung epithelium of *Hmga2*^*−/−*^ mice from 2.7% to 7.5% (*P* <0.01; *n* = 3), supporting the reduction of epithelium when compared with WT mice. However, since this increase of apoptosis cannot account for the drastic reduction of lung epithelium after *Hmga2*-KO, we examined lung epithelium differentiation by analyzing the expression of proximal, *Scgb1a1*, and distal, *Sftpc*, epithelial markers in the embryonic lung of WT and *Hmga2*-KO mice (Additional file 1: Figure S1B). Whereas *Scgb1a1* expression did not change significantly, *Sftpc* expression was reduced after *Hmga2*-KO, suggesting defects in distal epithelium differentiation. These defects were confirmed by immunostaining on sections of embryonic lung (Figure 3C,D, left) that showed reduced numbers of SFTPC-positive cells from 10.8% to 2.7% (*P* <0.001; *n* = 3) after *Hmga2*-KO when compared with the WT mice. These results were confirmed in lung explants after siRNA-mediated *Hmga2* knockdown (KD) (Additional file 2: Figure S2A). To further investigate these results, we performed immunostaining on sections of embryonic lung using antibodies specific for the distal epithelium progenitor cell marker sex determining region Y-box 9 (SOX9) and PCNA (Figure 3C,D, right). The number of SOX9-positive cells increased from 3.8% to 35.3% (*P* <0.001; *n* = 3) after *Hmga2*-KO when compared with WT mice. Interestingly, most of the SOX9-positive cells in the *Hmga2*-KO lung were proliferating as shown by PCNA co-staining. To summarize, *Hmga2*-KO increased cell proliferation more significantly in the mesenchyme of the embryonic lung, increased apoptosis in the epithelium, and reduced differentiation of the distal airway epithelium by altering the balance between progenitor cell renewal and differentiation. Taking together, our data support that *Hmga2* is required for proper distal epithelium differentiation.

### *Hmga2* knockout led to enhanced canonical WNT signaling

**Figure 4:**
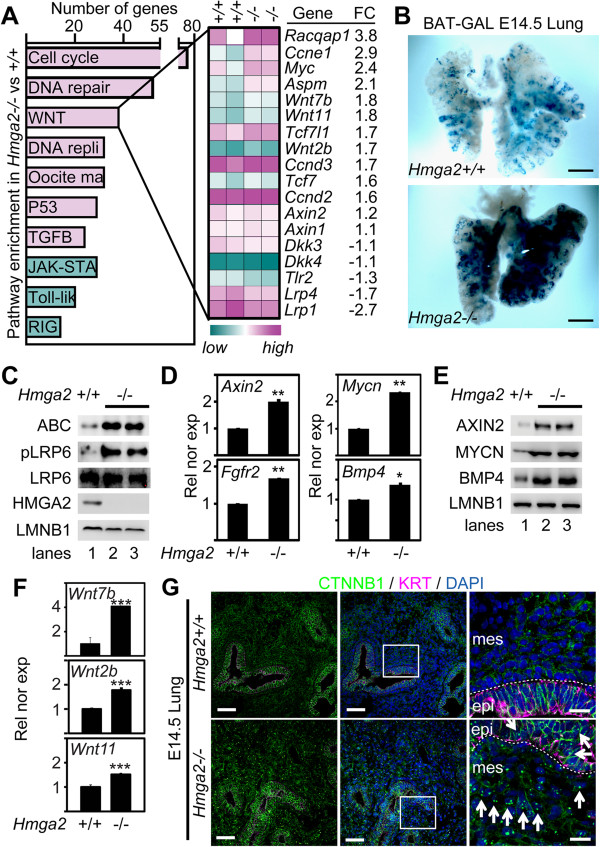
***Hmga2***^**−/− **^**mice embryonic lung showed enhanced canonical WNT signaling. (A)** Enriched cellular pathways (P <0.05) after Affymetrix microarray-based transcriptome analysis of *Hmga2*^−/−^ E18.5 lung when compared to *Hmga2*^+/+^. Left, Kyoto Encyclopedia of Genes and Genomes pathway analysis performed using DAVID bioinformatics tool. Green, reduced expression (fold change <1.2); magenta, increased expression (fold change ≥1.2); repli, replication; ma, maturation; JAK-STA, Jak-STAT signaling; Toll lik, Toll-like-receptor signaling; RIG, RIG-like-receptor signaling. Right, Heat map was done using DNAStar Arraystar 11.0 and represents expression of genes involved in the WNT signaling pathway in *Hmga2*^+/+^ and *Hmag2*^*−/−*^ E18.5 lung. Green, low expression; magenta, high expression; FC, fold change. **(B)***Hmga2*-KO enhanced beta-catenin/TCF/LEF reporter activity in E14.5 lungs of the BAT-GAL transgenic mice. Representative (*n* = 5) BAT-GAL staining (blue color) shows activated WNT signaling. Scale bars, 2 mm. **(C)***Hmga2*-KO increased levels of activated-beta-catenin (ABC) and phosphorylated LRP6 (pLRP6). Protein extracts from WT or *Hmga2*-KO E18.5 lungs were analyzed by western blot using the indicated antibodies. Lanes 2 and 3 are biological duplicates. **(D)***Hmga2*-KO enhanced expression of canonical WNT pathway markers. Expression analysis of the indicated genes as in Figure 2F. **(E)***Hmga2*-KO increased protein levels of canonical WNT pathway markers. Protein extracts were analyzed as in C using the indicated antibodies. **(F)***Hmga2*-KO enhanced expression of secreted WNT glycoprotein genes *Wnt7b*, *Wnt2b* and Wnt11. Expression analysis as in Figure 2F. **(G)***Hmga2*-KO increased nuclear localization of CTNNB1 in cells of the epithelium (epi) and the mesenchyme (mes) of the embryonic lung. Sections of E14.5 lung of WT and *Hmga2*-KO mice were analyzed as in Figure 2C using KRT- and CTNNB1- specific antibodies. Square shows details at higher magnification on the right. Nuclear staining with DAPI (blue). Arrows indicate nuclear CTNNB1. Dashed line shows epithelium-mesenchyme-boundary. Scale bars, 40 and 10 μm (right).

Comparison of *Hmga2*^−/−^ with *Hmga2*^*+/+*^ mice by Affymetrix microarray-based expression analysis of embryonic lung (Figure 4A) revealed an increased expression of cell-cycle-related genes, confirming our immunohistological results. We found that transcripts of genes that are either targets or positive regulators of canonical WNT signaling pathway increased in the embryonic lung of *Hmga2*-KO mice. Furthermore, expression of negative regulators of canonical WNT signaling was reduced in *Hmga2*-KO mice. These results indicate enhancement of canonical WNT signaling after *Hmga2*-KO that was further validated by increased activity of the beta-catenin/T-cell factor (TCF)/lymphoid enhancer factor (LEF) WNT-reporter (BAT-GAL) in embryonic lung of the BAT-GAL:*Hmga2*^*−/−*^ double transgenic mice when compared to BAT-GAL:*Hmga2*^*+/+*^ (Figure 4B). Western blot analysis of embryonic lung protein extracts (Figure 4C) showed increased levels of activated-beta-catenin (ABC) and phosphorylation of the WNT co-receptor low-density lipoprotein receptor-related protein 6 (LRP6) [18-20] after *Hmga2*-KO, confirming enhancement of canonical WNT signaling. However, beta-catenin (CTNNB1) has a dual role in both WNT signaling and cell adhesion processes. To confirm enhanced WNT signaling after *Hmga2*-KO, we analyzed the expression of canonical WNT targets, *Axin2, Mycn* (v-myc myelocytomatosis viral related oncogene), *Fgfr2* (fibroblast growth factor receptor 2) and *Bmp4* (bone morphogenetic protein 4) in the embryonic lung (Figure 4D). Expression of canonical WNT targets increased after *Hmga2*-KO when compared to WT mice, demonstrating elevated canonical WNT signaling. Western blot analysis of protein extracts from embryonic lung (Figure 4E) showed increased levels of canonical WNT targets after *Hmga2*-KO, confirming our expression analysis. Our results in the embryonic lung of *Hmga2*^*−/−*^ mice were supported by siRNA-mediated *Hmga2* depletion in the mouse lung epithelial cell line MLE-12 (Additional file 3: Figure S3), which increased the basal transcription of a co-transfected WNT-responsive luciferase reporter (*p3LEF-LUC*) more than twofold when compared to control siRNA (*siCtrl*)-transfected cells. Interestingly, forced expression of *Hmga2* reduced the basal transcription of *p3LEF-LUC*, thereby supporting an *Hmga2*-mediated negative regulation of WNT signaling.

Enhanced canonical WNT signaling is related to cell proliferation [21-23], correlating with our histological and molecular characterization of the *Hmga2*-KO embryonic lung. Nevertheless, we observed increased cell proliferation in the mesenchyme of embryonic lung after *Hmga2*-KO, although *Hmga2* expression is restricted to the distal epithelium of the embryonic lung. A plausible explanation for these two observations could be that enhanced WNT signaling after *Hmga2*-KO is induced in part by diffusible positive regulators of canonical WNT signaling. Indeed, our Affymetrix microarray-based expression analysis (Figure 4A) showed elevated expression of *Wnt11*, *Wnt7b* and *Wnt2b* in embryonic lung of *Hmga2*^*−/−*^ mice when compared to *Hmga2*^*+/+*^. These results were confirmed by qRT-PCR-based expression analysis (Figure 4F). Furthermore, immunostaining on sections of embryonic lung using CTNNB1-specific antibody (Figure 4G) showed increased translocation of CTNNB1 from the cytoplasm into the nucleus in cells of both the epithelium and the mesenchyme after *Hmga2*-KO, demonstrating elevated canonical WNT signaling in both tissues and explaining hyperproliferation and expansion of the mesenchyme after *Hmga2*-KO.

**Figure 5:**
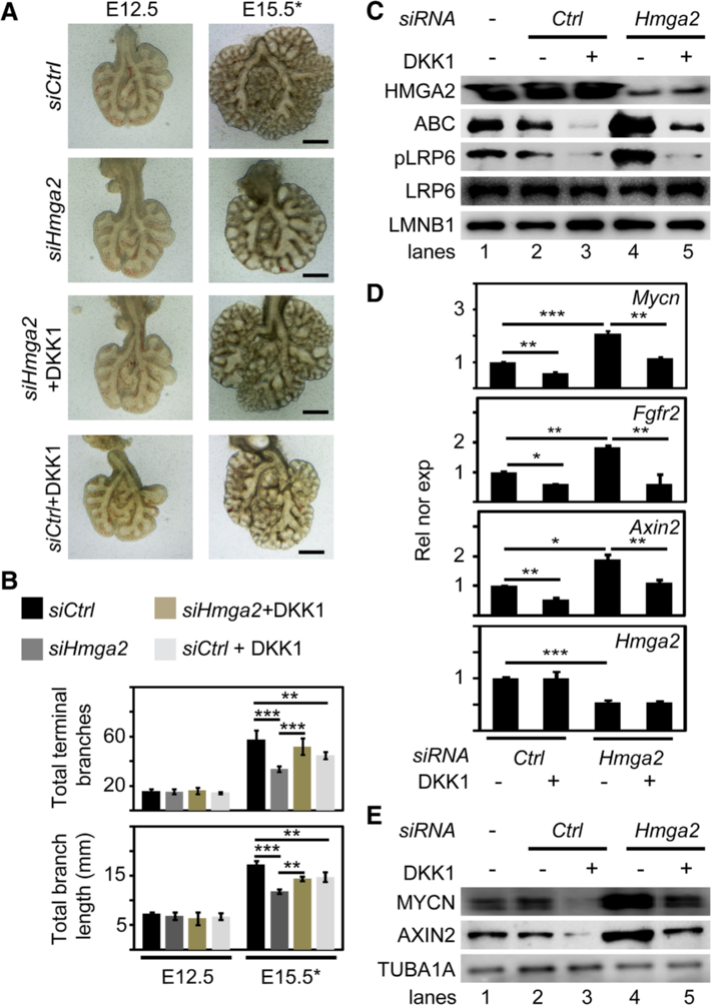
**Inhibition of canonical WNT signaling partially rescued the *Hmga2 *loss-of-function phenotype. (A)** Embryonic lung explants were cultured as in Additional file 4: Figure S4A. Explants were treated with control (*siCtrl*) or *Hmga2*-specific siRNAs (*siHmga2*) together with the canonical WNT inhibitor DKK1 for 72 h till stage E15 equivalent (E15*). Scale bars, 500 μm. **(B)** Terminal branches (top) and total branch length (bottom) were quantified at E12.5 and E15.5* Embryonic lung explants were treated as in A. Error bars show the SEM (*n* = 6). ***P <0.001; **P <0.01; *P <0.05 after one-way analysis of variance. **(C)** Inhibition of canonical WNT signaling antagonized the effect of *Hmga2*-LOF. Protein extracts from the lung explants treated as in A were analyzed by western blot using the indicated antibodies. LMNB1 was used as loading control. **(D)** Expression analysis of the indicated genes by quantitative RT-PCR in embryonic lung explants that were treated as in A. Relative expression normalized to *Tuba1a*. Error bars show the SEM (*n* = 3). Asterisks as B. **(E)** Protein extracts from the lung explants treated as in A were analyzed by western blot using the indicated antibodies. TUBA1A was used as loading control.

To confirm the causal involvement of canonical WNT signaling in mediating the effect of *Hmga2*-LOF, we used an organ explant culture system that mimics the normal embryonic lung development (Additional file 4: Figure S4A,B) [24]. Depletion of *Hmga2* in these organ explants by siRNA treatment (Additional file 4: Figure S4C,D) led to a marked impairment in branching morphogenesis when compared to explants exposed to control siRNA (*siCtrl*) (Figure 5A). These defects during the formation of the bronchial and respiratory tree after *Hmga2*-KD were validated by quantification of the total number of terminal branches and branch length (Figure 5B). When E12.5 lungs were cultured for 72 hours in the presence of a *Hmga2*-specific siRNA (*siHmga2*), the total number of terminal branches was reduced from 58 (*siCtrl*) to 34 (*P* <0.001; *n* = 6), and the branch length from 17 mm (*siCtrl*) to 12 mm (*P* <0.001; *n* = 6). However, treatment of the explants with the secreted canonical WNT inhibitor dickkopf homolog 1 (DKK1) [25] antagonized the defects caused by *siHmga2* (Figure 5A,B), reconstituting the total number of terminal branches to 51 (*P* <0.01; *n* = 6) and the branch length to 14 mm (*P* <0.01; *n* = 6). DKK1 treatment alone also affected branching morphogenesis by blocking WNT signaling as previously described from experiments in embryonic lung explants [26]. Western blot analysis of protein extracts from embryonic lung explants showed increased levels of ABC and phosphorylated LRP6 after *Hmga2*-KD (Figure 5C, lane 4), demonstrating enhanced canonical WNT signaling that was antagonized after DKK1 treatment (Figure 5C, lane 5). The partial rescue of the *Hmga2*-LOF phenotype achieved by DKK1-mediated block of WNT signaling was validated by expression analysis of canonical WNT targets (Figure 5D) and by western blot analysis of protein extracts from embryonic lung explants (Figure 5E). Our data demonstrated the causal involvement of increased WNT signaling in mediating the effect of *Hmga2*-LOF, suggesting a role of *Hmga2* in negative regulation of WNT signaling that was supported by reduced activity of the WNT-responsive *p3LEF-LUC* reporter after *Hmga2* over-expression in MLE-12 cells (Additional file 3: Figure S3).

### HMGA2 directly activates *Gata6* expression

**Figure 6:**
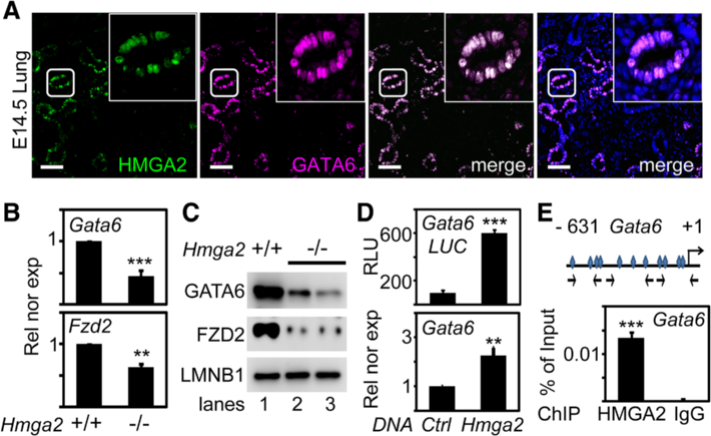
**HMGA2 directly regulates *Gata6 *and thereby modulates *Fzd2 *expression. (A)** HMGA2 and GATA6 co-localized in the nuclei of the same cells of the distal lung epithelium. Fluorescence microscopy of embryonic lung sections (E14.5) after double immunostaining using HMGA2- and GATA6-specific antibodies. Nuclear staining with DAPI (blue). Square shows details at higher magnification. Scale bars, 40 μm. **(B)***Hmga2*-KO decreased expression of *Gata6* and its downstream target gene *Fzd2*. Expression analysis of the indicated genes as in Figure 4D. Rel nor exp, relative expression normalized to *Tuba1a*. Data are presented as mean ± SEM (*n* = 4). ***P <0.001; **P <0.01 after one-way analysis of variance. **(C)** Protein extracts from WT (+/+) or *Hmga2*^*−/−*^ embryonic lung (E18.5) were analyzed as in Figure 4C using the indicated antibodies. **(D)***Hmga2* over-expression increased *Gata6* transcription. Top, Luciferase reporter assays of MLE-12 cells transiently transfected with a *Gata6*-*Luc* reporter plasmid and control (*Ctrl,* empty vector) or *Hmga2* expression construct. RLU, relative light units. Bottom, *Gata6* expression was monitored by quantitative RT-PCR in MLE-12 cells treated as above. Rel nor exp, relative expression normalized to *Gapdh*. Data are presented as mean ± SEM (*n* = 4) and asterisks as in B. **(E)** Endogenous HMGA2 binds to the endogenous *Gata6* promoter. Top, *in silico* analysis of the *Gata6* gene (-631 to +1 base pairs relative to transcription initiation site) revealed several HMGA2 binding elements (squares). Arrows, position of the primers used for ChIP. Bottom, ChIP of the *Gata6* promoter using HMGA2-specific antibody or IgG (negative control). ChIP, chromatin-immunoprecipitation assay; IgG, immunoglobulin G. Data are presented as mean ± SEM (*n* = 4) and asterisks as in B.

GATA6 is a transcription factor that is essential for branching morphogenesis and inhibits canonical WNT signaling in the distal epithelium of the lung by transcriptional activation of its downstream target, the canonical WNT-beta-catenin pathway antagonist *Fzd2*[11,27]. The defects observed after *Hmga2*-LOF were similar to the *Gata6*-LOF phenotype (Additional file 2: Figure S2A,B) [9]. Therefore, we investigated whether the enhancement of canonical WNT signaling after *Hmga2*-LOF could involve *Gata6* and *Fzd2*. Immunostaining on sections of embryonic lung (E14.5) using HMGA2- and GATA6-specific antibodies (Figure 6A) showed the presence of both proteins in the same cells of the distal lung endoderm. Interestingly, expression analysis in embryonic lung showed that *Gata6-* and *Fzd2*-transcripts decreased after *Hmga2*-KO when compared to WT mice (Figure 6B). Consistently, siRNA-mediated *Hmga2*-LOF reduced *Gata6* and *Fzd2* expression in embryonic lung explants (Additional file 2: Figure S2B). Western blot analysis of protein extracts from embryonic lung (Figure 6C) showed reduced levels of GATA6 and FZD2 after *Hmga2*-KO, supporting our expression analysis. To investigate the effect of *Hmga2* gain-of-function (GOF) on *Gata6* expression, MLE-12 cells were transiently transfected with *Hmga2* and a plasmid containing the luciferase (*Luc*) reporter gene under the control of the *Gata6* promoter (Figure 6D, top). Forced expression of *Hmga2* increased more than fivefold the basal transcription of the *Gata6-Luc* reporter. In addition, expression of endogenous *Gata6* increased more than twofold after *Hmga2* transfection (Figure 6D, bottom). Consistently, *in silico* analysis of the murine *Gata6* gene (Figure 6E, top) revealed several HMGA2 binding elements near the transcription initiation site. Moreover, chromatin immunoprecipitation assay (ChIP) in MLE-12 cells (Figure 6E, bottom) showed a direct association of endogenous HMGA2 to the endogenous *Gata6* promoter. Thus, we conclude that HMGA2 directly activates *Gata6* gene expression, and the enhanced WNT signaling caused by *Hmga2*-LOF could also be mediated by a reduction of the WNT signaling antagonizing proteins GATA6 and FZD2.

**Figure 7:**
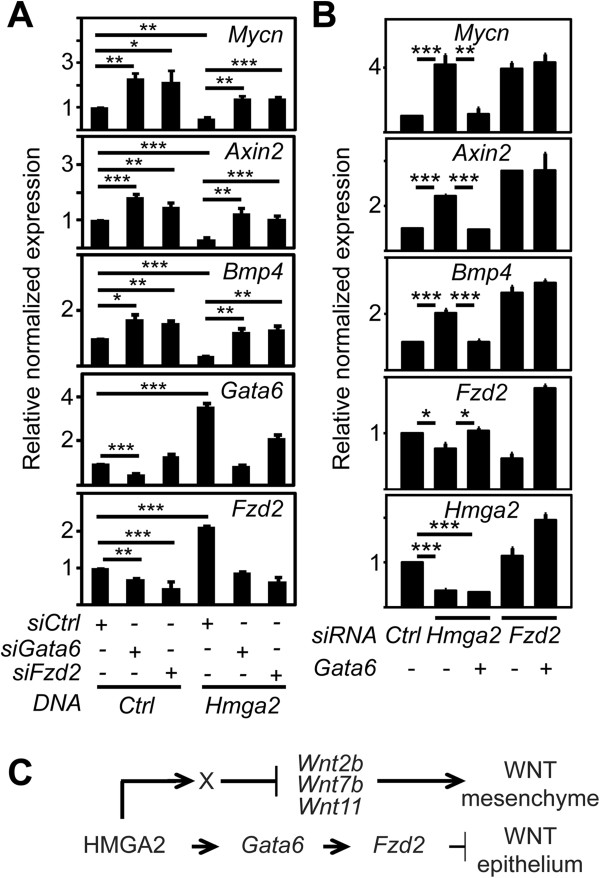
***Hmga2 *acts upstream of *Gata6 *in WNT signaling regulation. (A)***Gata6* and *Fzd2* are required for the effect of *Hmga2* GOF on expression of WNT targets. Expression analysis of the indicated genes by quantitative RT-PCR in MLE-12 cells that were transfected with either control (*Ctrl*) or *Gata6*- or *Fzd2*-specific siRNA and *Hmga2* expression plasmid as indicated. Data are presented as mean ± SEM (*n* = 4). ***P <0.001; **P <0.01; *P <0.05 after one-way analysis of variance. **(B)***Gata6-*GOF rescued the effect of *Hmga2*-LOF on expression of WNT targets. Expression analysis of the indicated genes by quantitative RT-PCR in MLE-12 cells that were transfected with either control (*Ctrl*) or *Hmga2*- or *Fzd2*-specific siRNA and *Gata6* expression plasmid as indicated. Data are presented as mean ± SEM (*n* = 4) and asterisks as in A. **(C)** Model. *Hmga2* regulates WNT signaling at different points of the pathway. Regulation of the secreted WNT glycoproteins (*Wnt2b*, *Wnt7b* and *Wnt11*) mediates the paracrine effect on the mesenchyme of the embryonic lung. In addition, HMGA2-mediated regulation of *Gata6* is a key process in fine-tuning the activity of canonical WNT signaling in developing airway epithelium. X, unknown transcription factor.

To show the requirement of *Gata6* in *Hmga2*-mediated regulation of WNT signaling, we transfected MLE-12 cells with *Hmga2* after siRNA-mediated *Gata6*- or *Fzd2*-depletion (Figure 7A). Expression analysis showed that *Hmga2*-GOF increased the levels of *Gata6* and *Fzd2* but reduced the levels of canonical WNT targets *Mycn*, *Axin2* and *Bmp4*, confirming a role of *Hmga2* in inhibition of canonical WNT signaling. Interestingly, *Gata6*- or *Fzd2*-depletion antagonized the effect of *Hmga2-*GOF on both groups of markers analyzed, indicating the requirement of *Gata6* and *Fzd2* for the negative regulation of WNT signaling mediated by *Hmga2*.

To determine the causal involvement of *Gata6* in *Hmga2*-mediated regulation of WNT signaling, we transfected MLE-12 cells with *Gata6* after siRNA-mediated *Hmga2*- or *Fzd2*-depletion (Figure 7B and Additional file 3: Figure S3B). Expression analysis showed that *Hmga2*- and *Fzd2*-LOF enhanced the expression of canonical WNT targets, supporting our previous expression analysis in the embryonic lung of *Hmga2*-KO mice and as expected from WNT signaling antagonizing genes. Interestingly, *Gata6* transfection compensated the effect of *Hmga2*-LOF, but not of *Fzd2*-LOF. Our data indicate that *Gata6* acts downstream of *Hmga2* and upstream of *Fzd2* in negative regulation of WNT signaling (Figure 7C).

## Discussion and conclusions

We showed that *Hmga2* is expressed in the embryonic mouse lung at the distal airways. *Hmga2* mRNA levels were high during early stages of lung development, in which cells are undifferentiated, and decreased as lung development progressed, coincident with cell differentiation. Interestingly, we detected a slight increase of *Hmga2* expression at E18.5 that matches with the establishment of a bipotent progenitor cell population in the distal epithelium [28]. Our data correlate with previous reports where HMGA2 was shown to be present at high levels in various undifferentiated tissues during embryonic development and in strongly reduced levels in the corresponding adult tissues [12-15].

*Hmga2*-KO induces a pygmy phenotype due to reduced expression of insulin-like growth factor 2 mRNA binding protein 2 (*Igf2bp2*) [15,16,29]. Prior to our study, the lung of *Hmga2*^*−/−*^ mice had not been analyzed. Detailed analysis of *Hmga2*^*−/−*^ mice showed that *Hmga2* is required for distal epithelium differentiation during embryonic lung development. *Hmga2*-KO led to enhanced canonical WNT signaling due to an increase of secreted WNT glycoproteins as well as a reduction of the WNT signaling antagonizing proteins GATA6 and FZD2, thereby supporting that *Hmga2* regulates WNT signaling at different points of the pathway (Figure 7C). The causal involvement of canonical WNT signaling in mediating the effect of *Hmga2*-LOF was demonstrated by the DKK1-induced rescue of *Hmga2*-LOF in embryonic lung explants (Figure 5A-E). HMGA2-mediated regulation of *Gata6* seems to be a key process in fine-tuning the activity of canonical WNT signaling in airway epithelium. The sequential order of events suggested in our model (Figure 7C) in which *Hmga2* acts upstream of *Gata6* is strongly supported by the fact that HMGA2 directly regulates *Gata6* (Figure 6A-E) as well as by the *Gata6*-mediated rescue experiments of *Hmga2*-LOF in MLE-12 cells (Figure 7B). Since HMGA2 regulates the transcription of its target genes by modulating the chromatin structure and by recruiting other proteins to the transcription regulatory complex [12], the scope of our future work will be to investigate in detail the mechanism of HMGA2-mediated transcriptional regulation of the *Gata6* promoter.

*Hmga2*-KO increased cell proliferation not only in the lung epithelium, where *Hmga2* is expressed, but also in the mesenchyme, suggesting a paracrine effect that could be explained by increased expression of the secreted components of WNT signaling. Since HMGA2 is known to activate transcription, the increased expression of *Wnt2b*, *Wnt7b* and *Wnt11* after *Hmga2*-KO suggest the participation of a transcription inhibitor that could block the expression of these secreted components of WNT signaling and whose expression could be regulated by HMGA2 (Figure 7C). Identification of this unknown mediator of HMGA2 will be the scope of future studies. Interestingly, analysis of the *Wnt7b* promoter showed that deletion of the region between -1,005 bp and -829 bp relative to the second transcription start site significantly increased the basal transcription activity of a *Wnt7b*-luciferase reporter [30], suggesting that the binding element of a putative transcription inhibitor was deleted in this construct.

The phenotypes of *Hmga2*- and *Gata6*-LOF in embryonic lung explants are very similar (Additional file 2: Figure S2A,B) [9]. However, the milder phenotype observed in the embryonic lung of *Hmga2*-KO mice when compared either with the *Gata6*-KO or the phenotype induced after *Hmga2*-KD in embryonic lung explants might be explained by redundancy in the function between *Hmga2* and *Hmga1*, another member of the HMG protein family. *Hmga1* transcript was reduced after *Hmga2*-LOF (Additional file 4: Figure S4C) but not affected in the *Hmga2*-KO mice (Additional file 1: Figure S1B). *Hmga1* might compensate the *Hmga2*-KO, thereby avoiding lethality at early embryonic stages, as is the case after *Gata6*-KO [31,32], or soon after birth due to defects in the lung, as is the case after lung epithelium-specific ablation of *Gata6*[11]*.* In addition, the expansion of the mesenchyme in the embryonic lung after *Hmga2*-KO and the apparent increase of epithelium in embryonic lung explants after *Hmga2*-KD might be explained by the differences of both LOF systems. In the transgenic approach, *Hmga2*-LOF takes place soon after fertilization and affects lung development from the initial stages of lung bud formation; in the explant culture, the LOF starts at E12.5, thereby reducing the rather indirect effect on the mesenchyme and making the effect on the epithelium more dominant. Analysis of the lungs in inducible and conditional double transgenic mice (*Hmga2*^*−/−*^:*Hmga1*^*−/−*^) would test these hypotheses and should be the scope of future studies.

*Hmga2* expression is positively regulated by transforming growth factor beta 1 signaling [33]. In addition, our data show that *Hmga2* antagonizes canonical WNT signaling. Therefore, it will be of interest to determine a potential opposing effect between these two signaling pathways in establishing the proximal-distal axis during branching morphogenesis and lung epithelium differentiation in the developing lung. *Hmga2* might play a crucial role on the interplay between these signaling pathways.

Organ regeneration requires a proper balance between self-renewal and differentiation of tissue-specific progenitor cells. Canonical WNT signaling has been implicated in different regenerative processes including zebrafish tail regeneration, zebrafish cardiac regeneration and expansion of anterior heart field progenitors in mammals [34,35]. Moreover, canonical WNT signaling is activated upon lung epithelial regeneration, and enhanced WNT activity caused by lung epithelium-specific ablation of *Gata6* led to a premature and increased number of BASCs [11]. BASCs represent one of several regional progenitor cell populations in the adult lung and are responsible for regeneration of bronchiolar and alveolar epithelium during homeostatic turnover and in response to injury [4,36]. Our study suggests the possible role of *Hmga2* in the adult lung controlling the balance between BASC expansion and differentiation. Our results are the starting point for future studies in which the relevance of *Hmga2*-mediated regulation of WNT signaling might be investigated in the adult lung within the context of proper balance between differentiation and self-renewal of lung stem/progenitor cells and lung regeneration during both homeostatic turnover and repair after injury. Characterization of the regulatory mechanisms controlling the proper balance between expansion and differentiation of lung stem/progenitor cells will have a profound impact on our understanding and treatment of lung disease.

## Methods

### Animal experiments

Mouse work was performed in compliance with the German Law for Welfare of Laboratory Animals. The permission to perform the experiments presented in this study was obtained from the Regional Council (Regierungspräsidium in Darmstadt, Germany). The numbers of the permissions are IVMr46-53r30.03.MPP04.12.02 and IVMr46-53r30.03.MPP06.12.01. Animals were killed for scientific purposes according to the law mentioned above, which complies with national and international regulations.

C57BL/6 and *Hmga2*^*+/−*^ mice (stock # 002644, Jackson Laboratories) [37] were obtained from Charles River Laboratories (Germany) at 5 to 6 week of age. BAT-GAL transgenic reporter mice (also called beta-catenin/TCF/LEF reporter transgenic mice) were obtained as a gift from Prof. Stefan Liebner [38]. BAT-GAL:*Hmga2*^*−/−*^ double transgenic embryos were obtained by crossing heterozygous BAT-GAL:*Hmga2*^*+/−*^ mice. Animals were housed and bred under controlled temperature and lighting (12/12-hour light/dark cycle), fed with commercial animal feed and water *ad libitum*. All experiments were performed with mouse embryonic lungs. Timed-pregnant C57BL/6 WT mice were killed at indicated time points; embryonic lungs were isolated according to standard methods and whole-mount *in situ* hybridization was performed as described [39] with minor modifications. Briefly, to synthesize digoxigenin-labeled RNA probes, *pcDNA3-mHmga2* plasmid (gift from Prof. Peter Grouse) [40] was linearized, and UTP-digoxigenin (Roche) substituted antisense RNA probes were transcribed with T7 RNA polymerase. Sense RNA probes as negative control were transcribed with SP6 RNA polymerase.

### Embryonic lung explants culture

Lungs of timed-pregnant C57BL/6 WT mice were dissected from the embryos at E12.5 and cultured for 72 hours till E15.5 equivalent (E15.5*) as previously reported [24]. The explants were treated with 3 μM siRNAs against *Hmga2* (Applied Biosystems, Silencer Select siRNAs, Assay ID s67600), scrambled siRNA (negative control, *Ctrl*) (Sigma, MISSION siRNA Universal Negative Control, SIC001) or 200 nM of mouse recombinant DKK1 (R&D Systems, 5897-DK-010) following a similar protocol as previously described [9,41-44]. The siRNA and protein treatments were renewed every 24 hours. After 72 hours, the lungs were checked for morphological changes by standard microscopy techniques and harvested for RNA (QIAGEN RNeasy Micro Kit) and protein isolation. The images were used to determine the total number of terminal bud branches and for quantification of total branch length as described [45].

### Cell culture transfection assays

Mouse lung epithelial cells (MLE-12, ATCC CRL-2110) were cultured following the supplier’s instructions. MLE-12 cells were transiently transfected either with 40 nM *siCtrl* (negative control; AM4611, Ambion), 40 nM *siGata6* (L-065585-00, Dharmacon), 20 nM *siFzd2* (s81164, Applied Biosystems), 20 nM si*Hmga2* (s67600, Applied Biosystems) and/or *pcDNA 3.1(A)-Hmga2-myc/His* or *pCMV6-entry-Gata6-flag/myc* or *pBL* (Ctrl) as indicated using Lipofectamine 2000 transfection reagent (Invitrogen) at a ratio of 1:2 DNA:Lipofectamine. Cells were harvested 48 hours later for further analysis.

The proximal 631 bp *Gata6* promoter was amplified and cloned into the pGL4basic vector to generate *pGL4-Gata6* promoter luciferase vector. Dual-luciferase reporter assays (Promega) were performed as described [46] following transient transfection of MLE-12 cells in 96-well plates with 20 nM effector siRNAs and a total of 100 ng DNA per well, containing 15 ng effector plasmid, 15 ng *pGL4-Gata6* promoter or *p3LEF-LUC* luciferase reporter plasmid, 1 ng *Renilla* luciferase reporter plasmid and 69 ng pBluescript. Each sample was performed in triplicate. Each experiment was repeated at least three times.

### Affymetrix microarrays, quantitative PCR and ChIP assays

Total RNA was isolated with RNeasy® plus mini kit (Qiagen) and quantified using a spectrophotometer. Affymetrix microarray-based transcriptome analysis of *Hmga2*^−/−^ and *Hmga2*^*+/+*^ embryonic lung (E18.5) was performed and analyzed as described [47]. Kyoto Encyclopedia of Genes and Genomes pathway enrichment based analysis of dysregulated pathways in *Hmga2*^−/−^ versus *Hmga2*^*+/+*^ was done using DAVID software [48] and generation of fold change and Heat map were performed using DNAStar Arraystar 11.0. The data discussed in this publication have been deposited in NCBI’s Gene Expression Omnibus [49] through accession number [GEO:GSE55340] (http://www.ncbi.nlm.nih.gov/geo/query/acc.cgi?acc=GSE55340).

The High Capacity Reverse Transcription kit (Applied Biosystems) was used for synthesis of cDNA from total RNA. Quantitative real-time PCR reactions were performed using SYBR® Green on the Step One plus Real-time PCR system (Applied Biosystems). The PCR results were normalized with respect to the housekeeping gene for tubulin alpha 1a (*Tuba1a*) or glyceraldehyde-3-phosphate dehydrogenase (*Gapdh*).

ChIP analysis of the mouse *Gata6* promoter was performed as described [50] with slight modifications. Briefly, MLE-12 cells were cross-linked by 1% formaldehyde for 10 minutes, lysed, and sonicated with Diagenode Biorupter to an average DNA length of 500 to 600 bp. After isolation, the soluble chromatin was immunoprecipitated with immunoglobulin G (control, Santa Cruz) or HMGA2-specific antibody (sc-30223; Santa Cruz Biotechnology). Reverse cross-linked immunoprecipitated chromatin was subjected to qPCR using the primers listed in Additional file 5: Table S1.

### Immunohistochemistry

For paraffin-embedded mouse embryonic lung tissue, lungs were fixed overnight in 1% paraformaldehyde at 4°C, dehydrated over a graded series of alcohol, and paraffin embedded. Sections of 4 μm were prepared on a microtome (Leica). Antigen retrieval was performed by microwave heating for 8 minutes using 1 mM EDTA (pH 8) or 1 mM citrate buffer (pH 6). For cryosections of mouse lung tissue, lungs were harvested and embedded in polyfreeze tissue freezing medium (Polysciences). Sections of 10 μm were prepared on a cryostat (Leica). Sections were post-fixed in 4% paraformaldehyde for 10 minutes. Antibody staining was performed following standard procedures. All incubations and washes were performed with histobuffer containing 3% bovine serum albumin and 0.2% Triton X-100 in 1× phosphate-buffered saline, pH 7.4. Non-specific binding was blocked by incubating with donkey serum and histobuffer (1:1 (v/v) ratio) for 45 to 60 minutes. The sections were then incubated with primary and secondary antibodies for 60 minutes followed by nuclear staining. The sections were examined with a confocal microscope (Zeiss) or fluorescent microscope (Leica). Antibodies used were specific against HMGA2 (BioCheck), LMNB1 (Santa Cruz), GATA6 (R&D system), cadherin 1 (Abcam), VIM-Cy3 (Sigma), PCNA (Santa Cruz), MKi67 (Abcam), KRT (Dako and Sigma), Pro-SFTPC (Millipore), SOX9 (Santa Cruz), ABC (Millipore), CTNNB1 (Abcam), ACTA2-Cy3 (Sigma) and clCASP3 (Cell Signaling). Secondary antibodies used were Alexa 488, Alexa 633 and Alexa 555 (Invitrogen). DAPI (Invitrogen) were used as nuclear dye.

Paraformaldehyde-fixed and paraffin-embedded lung tissue sections were stained with hematoxylin and eosin and used for the lung morphology analysis. Figures were elaborated following a color scheme recommended to make them visible and easy to interpret by people with all types of color vision [51,52].

### Western blot

Western blotting was performed following standard protocols and using antibodies specific for HMGA2 (Biocheck and Santa Cruz), TUBA1A (Sigma), GATA6 (R&D system), FZD2 (Abcam), active-beta-catenin (ABC, Millipore), LRP6 (Cell signaling), phosphorylated LRP6 (Cell signaling), AXIN2 (Abcam), BMP4 (Millipore), MYCN (Santa Cruz) and LMNB1 (Santa Cruz). Immunoreactive proteins were visualized with the corresponding horseradish peroxide-conjugated secondary antibodies using the Super Signal West Femto detection solutions (Thermo Scientific). Signals were detected and analyzed with Luminescent Image Analyzer (Las 4000, Fujifilm).

### Statistical analysis

In total, the lungs of six *Hmga2*^*−/−*^ mice were analyzed using different techniques. The lungs of six *Hmga2*^*+/+*^ were used as control because the heterozygote *Hmga2*^*+/−*^ mice presented a mild phenotype. With the exception of the Affymetrix array-based expression analysis, the experiments were performed at least three times and the samples in each experiment were analyzed in triplicates. The Affymetrix array-based expression analysis was performed one time using biological duplicates. Statistical analyses were performed using Excel Solver. All data are represented as mean ± SEM. One-way analyses of variance (ANOVA) were used to determine the levels of difference between the groups and *P* values for significance.

## Abbreviations

BASC: bronchioalveolar stem cells; bp: base pairs; ChIP: chromatin imunoprecipitation; GOF: gain of function; KD: knockdown; KO: knockout; LOF: loss of function; qRT-PCR: quantitative reverse transcription polymerase chain reaction; siRNA: short interfering RNA; WT: wild type; E15.5*: E15.5 equivalent.

## Competing interests

The authors declare that they have no competing interests.

## Authors’ contributions

Luc-assays, western blots, *in situ* hybridization and ChIPs were performed by IS; immunohistochemistry by IS and AM; RT-PCR by IS, GB, AM and AC; embryonic lung explants culture by AM, IS, MW and GC; Affymetrix microarray-based expression analysis by TB and AC; TB, MW, HACF, TBr, WS, SB and GC were involved in study design; GB and IS designed the study, analyzed data and wrote the manuscript. All authors discussed the results and commented on the manuscript. All authors read and approved the final manuscript.

## Authors’ information

All affiliations in Germany are members of the Universities of Giessen and Marburg Lung Center (UGMLC) and the German Center of Lung Research (DZL).

## Supplementary Material

Additional file 1: Figure S1 Characterization of embryonic lung in *Hmga2*^−/−^ mice. **(A)** Top, *Hmga2*^−/−^ mouse embryos at E18.5 were smaller than WT embryos. Main interval of the scale is equivalent to 1 cm. Bottom, *Hmga2*^−/−^ embryo showed 40% body wet weight reduction when compared to WT embryos. Data are represented as mean ± SEM (*n* = 3). ***P <0.001; **P <0.01; *P <0.05. **(B)** Expression analysis of the indicated genes by qRT-PCR in embryonic lung (E18.5) of WT and *Hmga2*^*−/−*^ mice. Rel nor exp, relative expression normalized to *Tuba1a*.; *Scgb1a1*, secretoglobin 1A1 also known as CC10; *Sftpc*, surfactant-associated protein C also known as SPC. Error bars, SEM (*n* = 4). Asterisks as in A. **(C)** Sections of embryonic lung (E18.5) of WT (+/+) and *Hmga2*^*−/−*^ mice were analyzed by confocal microscopy after double immunostaining using (left) MKI67- and CDH1-specific antibodies or (right) PCNA- and VIM-specific antibodies. Nuclear staining with DAPI (blue). Scale bars, 40 μm. Click here for file

Additional file 2: Figure S2 *Hmga2* and *Gata6* loss-of-function in embryonic lung explants led to a marked impairment of airway epithelial differentiation due to enhanced canonical WNT activity. **(A)***Hmga2*-KD in lung explants led to dilated airways and resulted in lung epithelial differentiation defects. Phenotype of *Gata6*-KD in lung explants was similar to the *Hmga2*-KD. Top, embryonic lungs were explanted and cultured until E15.5* as in Additional file 4: Figure S4A. Explants were treated with control (*siCtrl*), *Hmga2*- (*siHmga2*) or *Gata6-* (*siGata6*) specific *siRNAs*. Scale bars, 500 μm. Bottom, sections of treated explants were analyzed by confocal microscopy after immunostaining using either SFTPC- or with ABC-specific antibodies as indicated. Nuclear staining with DAPI (blue). ABC, activated-beta-catenin. Scale bars, 40 μm. **(B)***Hmga2* or *Gata6* knockdown enhanced expression of canonical WNT pathway markers and reduced *Fzd2* expression. Expression analysis of the indicated genes as in Additional file 4: Figure S4C. Gene expression normalized to *Tub1a1. Fzd2*, *frizzled homolog 2*; *Fgfr2*, *fibroblast growth factor receptor 2*. Error bars, SEM (*n* = 4). ***P <0.001; **P <0.01; *P <0.05. Click here for file

Additional file 3: Figure S3 *Hmga2* depletion increased the transcription of a WNT-responsive reporter whereas *Hmga2* forced expression reduced it. **(A)** Luciferase reporter assays of MLE-12 cells transiently transfected with a *p3LEF-LUC* reporter plasmid and control (*Ctrl*) or Hmga2 specific siRNA (siHmga2); or *Hmga2* expression construct; or treated with lithium chloride (LiCl, positive control). Error bars, SEM (*n* = 3). **(B)***Gata6-*GOF rescued the effect of *Hmga2*-LOF on expression of WNT targets. Expression analysis of the indicated genes by qRT-PCR in MLE-12 cells that were transfected with either control (*Ctrl*) or *Hmga2*- or *Fzd2*-specific siRNA and *Gata6* expression plasmid as indicated. Data are represented as mean ± SEM (*n* = 4). ***P <0.001; **P <0.01; *P <0.05. Click here for file

Additional file 4: Figure S4 *Hmga2* loss-of-function in embryonic lung explants led to a marked impairment of airway epithelial differentiation due to enhanced canonical WNT activity confirming the results obtained in *Hmga2*^*−/−*^ mice. **(A)** Schematic diagram of embryonic lung explant culture experiments. Mice embryonic lungs were explanted at E12.5, cultured for 72 hours until E15.5* and harvested for gene expression analysis and immunostaining. Scale bars, 500 μm. **(B)** Embryonic lung explant cultures mimic the normal embryonic lung development. Expression analysis of the indicated genes by qRT-PCR in embryonic lungs (E12.5, E15.5) and in explanted lungs (E15.5*) as in A. Rel nor exp, relative expression normalized to *Tub1a1*. Data are represented as mean ± SEM (*n* = 4). **(C)** s*iRNA*-mediated *Hmga2*-LOF was efficient in lung explants. Embryonic lungs were explanted and cultured until E15.5* as in A. Explants were treated with control (*siCtrl*) or *Hmga2*-specific (*siHmga2*) *siRNAs*. Expression of the indicated genes was analyzed by qRT-PCR. Rel nor exp, relative expression normalized to *Tub1a1*. Error bars, SEM (*n* = 4). ***P <0.001; *P <0.05. **(D)** Lung explants were treated with *siRNAs* as in C. Protein extracts of treated explants were analyzed by western blot using HMGA2-, ABC- or TUBA1A-specific antibodies. **(E)***Hmga2*-KD enhanced the activity of the beta-catenin/TCF/LEF reporter in lung explants of the BAT-GAL transgenic mice. Lung explants of BAT-GAL mice were cultured as in A. Explants were treated with control (*siCtrl*) or *Hmga2*-specific siRNAs (*siHmga2*) or with lithium chloride (LiCl, positive control). Beta-galactosidase staining was performed with the treated explants to detect activated WNT signaling (blue color). Scale bars, 500 μm. Click here for file

Additional file 5: Table S1 Oligonucleotide sequences used for real-time qPCR and ChIP. Click here for file
