# Supplementary figures and images for: Hmga2 is required for canonical WNT signaling during lung development
Source: BMC Biol. 2014 Mar 24;12:21. doi: 10.1186/1741-7007-12-21 (PMC4064517; doi:10.1186/1741-7007-12-21)

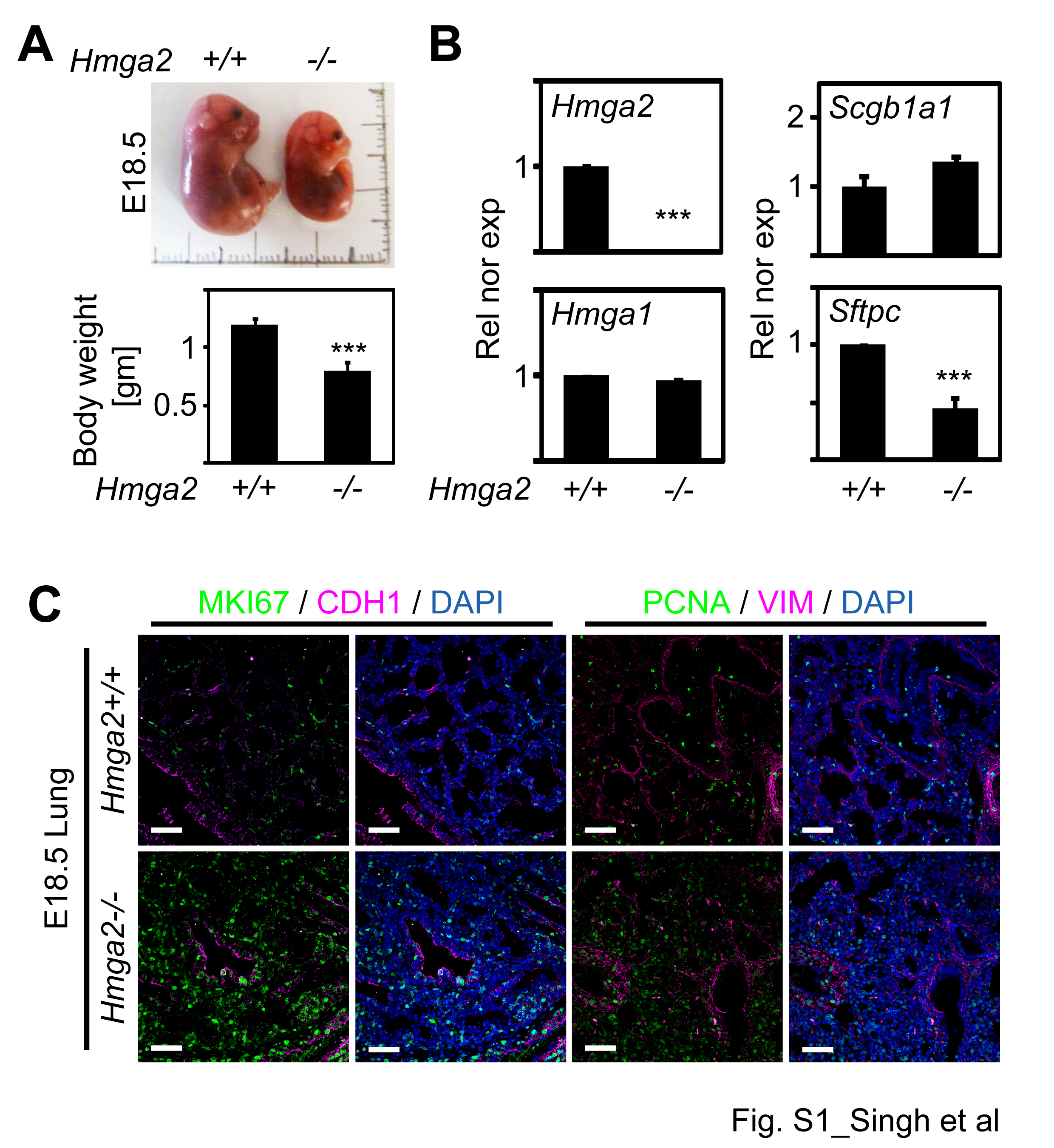

Supplement: Additional file 1: Figure S1 — Characterization of embryonic lung in Hmga2−/− mice. (A) Top, Hmga2−/− mouse embryos at E18.5 were smaller than WT embryos. Main interval of the scale is equivalent to 1 cm. Bottom, Hmga2−/− embryo showed 40% body wet weight reduction when compared to WT embryos. Data are represented as mean ± SEM (n = 3). ***P <0.001; **P <0.01; *P <0.05. (B) Expression analysis of the indicated genes by qRT-PCR in embryonic lung (E18.5) of WT and Hmga2 −/− mice. Rel nor exp, relative expression normalized to Tuba1a.; Scgb1a1, secretoglobin 1A1 also known as CC10; Sftpc, surfactant-associated protein C also known as SPC. Error bars, SEM (n = 4). Asterisks as in A. (C) Sections of embryonic lung (E18.5) of WT (+/+) and Hmga2 −/− mice were analyzed by confocal microscopy after double immunostaining using (left) MKI67- and CDH1-specific antibodies or (right) PCNA- and VIM-specific antibodies. Nuclear staining with DAPI (blue). Scale bars, 40 μm. [file 1741-7007-12-21-S1.tiff]

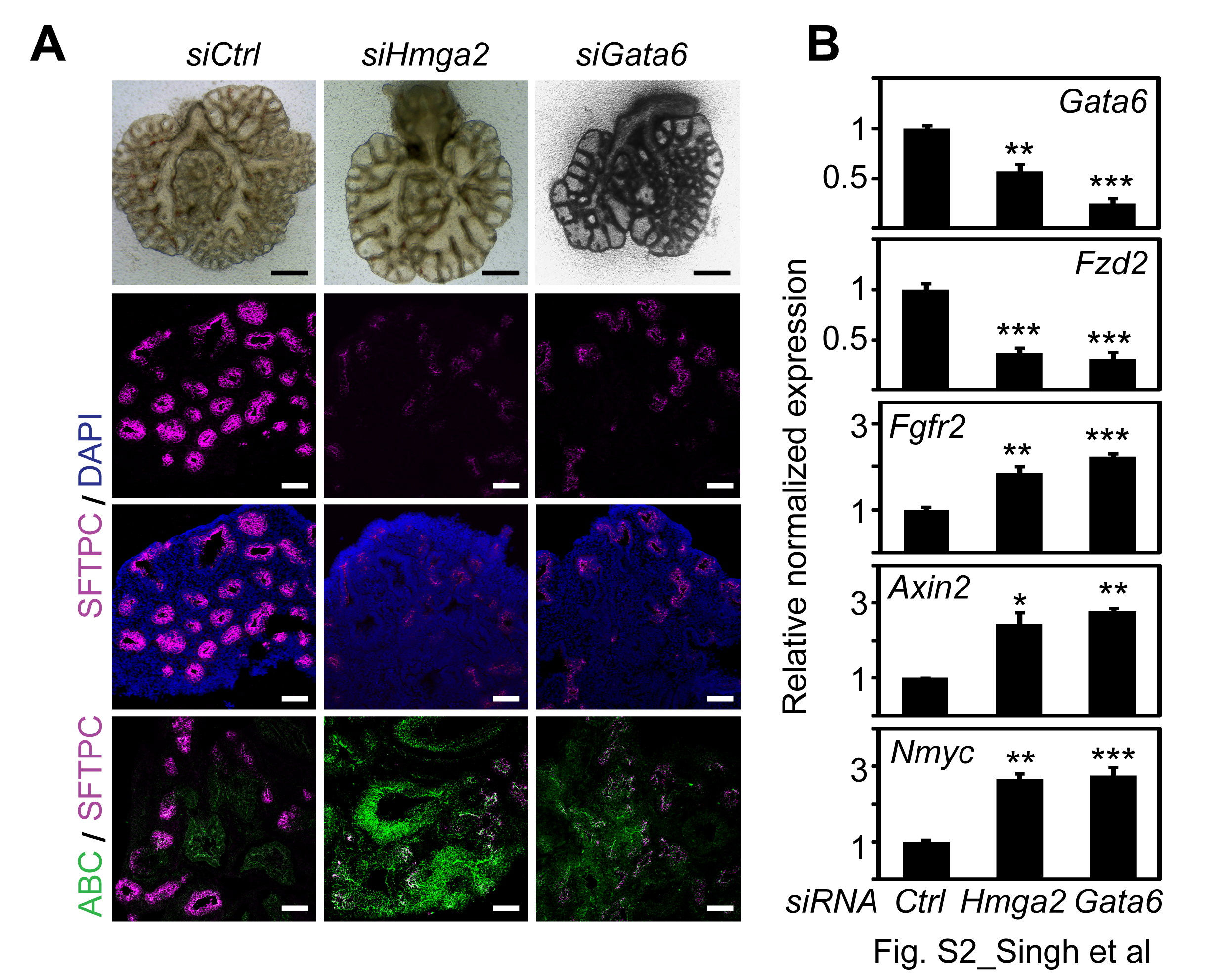

Supplement: Additional file 2: Figure S2 — Hmga2 and Gata6 loss-of-function in embryonic lung explants led to a marked impairment of airway epithelial differentiation due to enhanced canonical WNT activity. (A)Hmga2-KD in lung explants led to dilated airways and resulted in lung epithelial differentiation defects. Phenotype of Gata6-KD in lung explants was similar to the Hmga2-KD. Top, embryonic lungs were explanted and cultured until E15.5* as in Additional file 4: Figure S4A. Explants were treated with control (siCtrl), Hmga2- (siHmga2) or Gata6- (siGata6) specific siRNAs. Scale bars, 500 μm. Bottom, sections of treated explants were analyzed by confocal microscopy after immunostaining using either SFTPC- or with ABC-specific antibodies as indicated. Nuclear staining with DAPI (blue). ABC, activated-beta-catenin. Scale bars, 40 μm. (B)Hmga2 or Gata6 knockdown enhanced expression of canonical WNT pathway markers and reduced Fzd2 expression. Expression analysis of the indicated genes as in Additional file 4: Figure S4C. Gene expression normalized to Tub1a1. Fzd2, frizzled homolog 2; Fgfr2, fibroblast growth factor receptor 2. Error bars, SEM (n = 4). ***P <0.001; **P <0.01; *P <0.05. [file 1741-7007-12-21-S2.tiff]

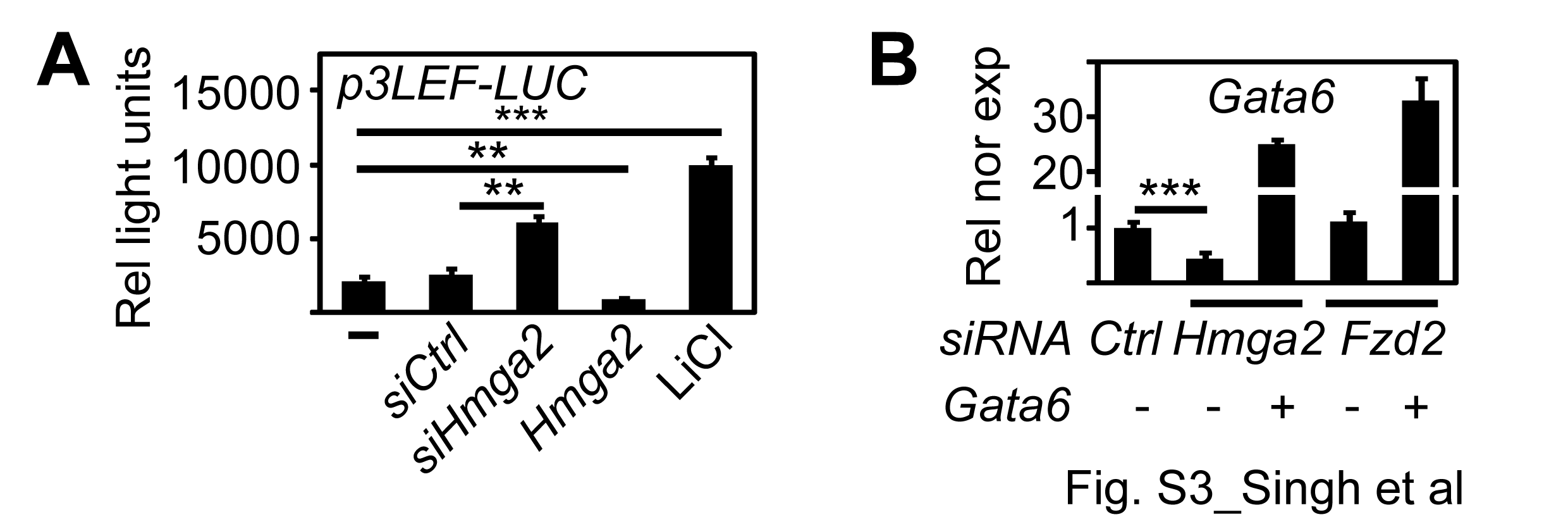

Supplement: Additional file 3: Figure S3 — Hmga2 depletion increased the transcription of a WNT-responsive reporter whereas Hmga2 forced expression reduced it. (A) Luciferase reporter assays of MLE-12 cells transiently transfected with a p3LEF-LUC reporter plasmid and control (Ctrl) or Hmga2 specific siRNA (siHmga2); or Hmga2 expression construct; or treated with lithium chloride (LiCl, positive control). Error bars, SEM (n = 3). (B)Gata6-GOF rescued the effect of Hmga2-LOF on expression of WNT targets. Expression analysis of the indicated genes by qRT-PCR in MLE-12 cells that were transfected with either control (Ctrl) or Hmga2- or Fzd2-specific siRNA and Gata6 expression plasmid as indicated. Data are represented as mean ± SEM (n = 4). ***P <0.001; **P <0.01; *P <0.05. [file 1741-7007-12-21-S3.tiff]

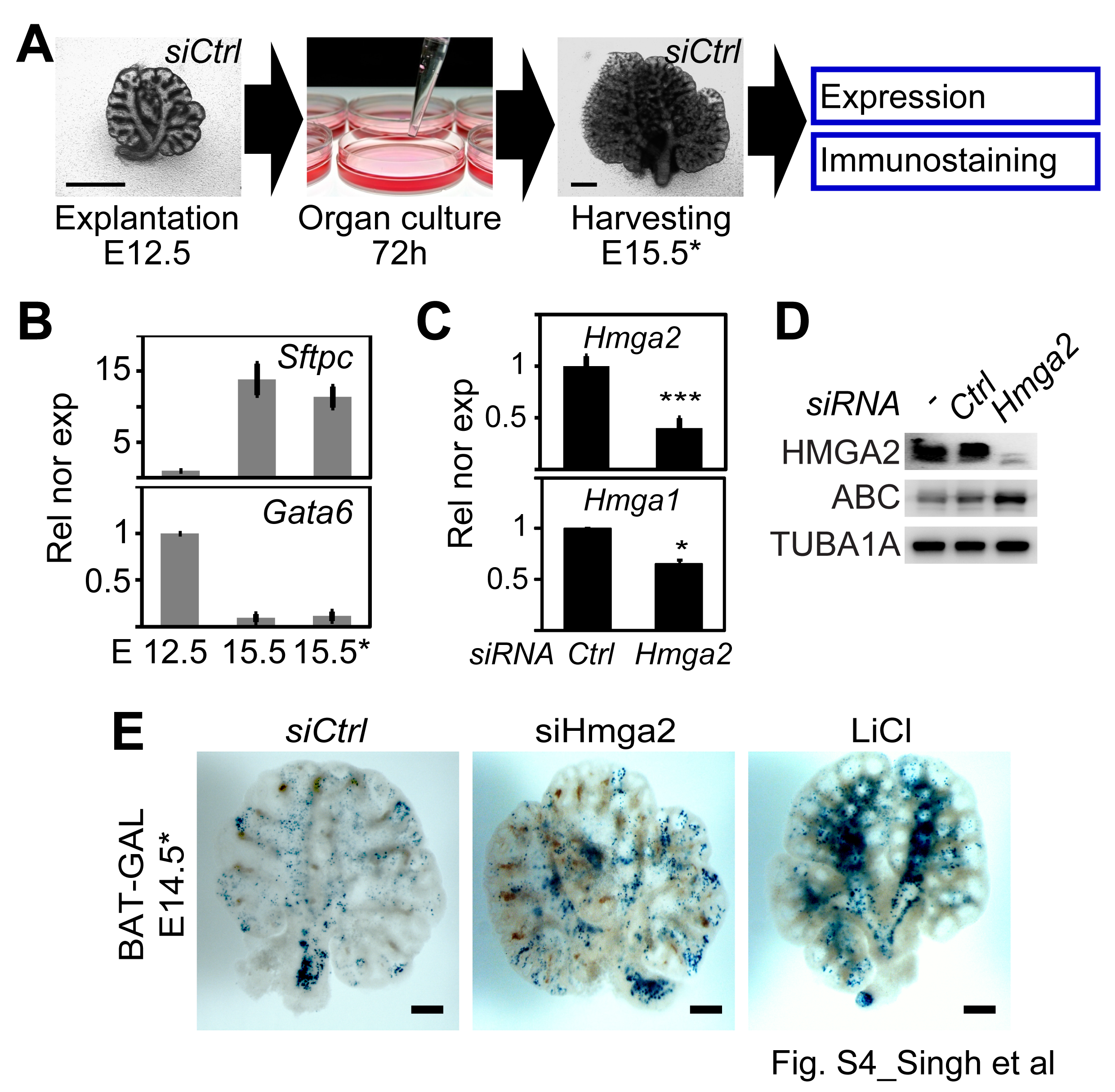

Supplement: Additional file 4: Figure S4 — Hmga2 loss-of-function in embryonic lung explants led to a marked impairment of airway epithelial differentiation due to enhanced canonical WNT activity confirming the results obtained in Hmga2 −/− mice. (A) Schematic diagram of embryonic lung explant culture experiments. Mice embryonic lungs were explanted at E12.5, cultured for 72 hours until E15.5* and harvested for gene expression analysis and immunostaining. Scale bars, 500 μm. (B) Embryonic lung explant cultures mimic the normal embryonic lung development. Expression analysis of the indicated genes by qRT-PCR in embryonic lungs (E12.5, E15.5) and in explanted lungs (E15.5*) as in A. Rel nor exp, relative expression normalized to Tub1a1. Data are represented as mean ± SEM (n = 4). (C) siRNA-mediated Hmga2-LOF was efficient in lung explants. Embryonic lungs were explanted and cultured until E15.5* as in A. Explants were treated with control (siCtrl) or Hmga2-specific (siHmga2) siRNAs. Expression of the indicated genes was analyzed by qRT-PCR. Rel nor exp, relative expression normalized to Tub1a1. Error bars, SEM (n = 4). ***P <0.001; *P <0.05. (D) Lung explants were treated with siRNAs as in C. Protein extracts of treated explants were analyzed by western blot using HMGA2-, ABC- or TUBA1A-specific antibodies. (E)Hmga2-KD enhanced the activity of the beta-catenin/TCF/LEF reporter in lung explants of the BAT-GAL transgenic mice. Lung explants of BAT-GAL mice were cultured as in A. Explants were treated with control (siCtrl) or Hmga2-specific siRNAs (siHmga2) or with lithium chloride (LiCl, positive control). Beta-galactosidase staining was performed with the treated explants to detect activated WNT signaling (blue color). Scale bars, 500 μm. [file 1741-7007-12-21-S4.tiff]
