# Supplementary material for: Hmga2 is required for canonical WNT signaling during lung development
Source: BMC Biol. 2014 Mar 24;12:21. doi: 10.1186/1741-7007-12-21 (PMC4064517; doi:10.1186/1741-7007-12-21)
Supplement: Additional file 5: Table S1 — Oligonucleotide sequences used for real-time qPCR and ChIP. [file 1741-7007-12-21-S5.docx]

**Supplemental table 1. Oligonucleotide sequences used for real-time quantitative PCR and ChIP.**

| **Gene** | **Primer sequence** | |
| --- | --- | --- |
| ***Hmga2*** | **Forward 1** | **5’GCAGCAGCAAGAGCCAACCTG** |
|  | **Reverse 1a** | **5’CTGCGGACTCTTGCGAGGATG** |
|  | **Reverse 1b** | **5’GTCTCTTCAGTCTCCTGAGCA** |
|  | **Reverse 1c** | **5’AAGCGATGAGCTCCTGCCCA** |
| ***Gapdh*** | **Forward** | **5’TGAGTATGTCGTGGAGTCTAC** |
|  | **Reverse** | **5’TGGACTGTGGTCATGAGCC** |
| ***Tuba1a*** | **Forward** | **5’CCGCGAAGCAGCAACCAT** |
|  | **Reverse** | **5’CCAGGTCTACGAACACTGCC** |
| ***Axin2*** | **Forward** | **5’GAGTAGCGCCGTGTTAGTGACT** |
|  | **Reverse** | **5’CCAGGAAAGTCCGGAAGAGGTATG** |
| ***Fgfr2**** | **Forward** | **5’GCTTCTCAGTGAGTTTTAATAACAGC** |
|  | **Reverse** | **5’GAATGATGCTGGGCTTTTGC** |
| ***Mycn**** | **Forward** | **5’TGTGTTGACATTAAGAATGTTGGTTTAC** |
|  | **Reverse** | **5’TTTCCAAGGTCATGGCAGAAC** |
| ***Bmp4**** | **Forward** | **5’CCCTTTCCACTGGCTGATCA** |
|  | **Reverse** | **5’GGGACACAACAGGCCTTAGG** |
| ***Wnt 2b*** | **Forward** | **5’TGTGTCAACGCTACCCAGAC** |
|  | **Reverse** | **5’TAGCATAGACGAACGCTGCC** |
| ***Wnt 11*** | **Forward** | **5’GCTGCGTCTGGAAGAAGCTAT** |
|  | **Reverse** | **5’AGTGGATAGGGAGAGTGCGG** |
| ***Wnt7b*** | **Forward** | **5’ GCATCCAAGGTCAACGCAAT** |
|  | **Reverse** | **5’CTCAGAGTCTCATGGTCCCTTTG** |
| ***Fzd2*** | **Forward** | **5’CCGCTCTTCGTATACCTGTTC** |
|  | **Reverse** | **5’CGGATGCGGAAGAGTGACA** |
| ***Gata6*** | **Forward** | **5’ATGGCGTAGAAATGCTGAGGG** |
|  | **Reverse** | **5’TGAGGTGGTCGCTTGTGTAG** |
| ***Hmga1*** | **Forward** | **5’CCAGTGAAGTGCCAACTCCGA** |
|  | **Reverse** | **5’CGGCACTGCGAGTGGTGATC** |
| ***Scgb1a1*** | **Forward** | **5’CTGAAGAGACTGGTGGATACC** |
|  | **Reverse** | **5’GTTTATTGCAAGAGGAAGGA** |
| ***Sftpc*** | **Forward** | **5’CGTGGTTGTGGTGGTGGTC** |
|  | **Reverse** | **5’GGATGCTCTCTGGAGCCATC** |
| ***Gata6***  **(ChIP)** | **Forward 1** | **5’CCCACGACCTGAGCATCCCG** |
|  | **Reverse 1** | **5’GAGGGACTCGCCCCCTCCTG** |
|  | **Forward 2** | **5’CCAGCTCCTTCCGAGCCAAGT** |
|  | **Reverse 2** | **5’GGGGCGCCCAGCTAAAGGAC** |
|  | **Forward 3** | **5’CCGGGGTGGACTCGCTCCTA** |
|  | **Reverse 3** | **5’GCCAGGCTGTGGGTCGGAAC** |
| ***Gata6 promoter***  **cloning** | **Forward** | **5’CCCACGACCTGAGCATCCCG** |
|  | **Reverse** | **5’GCCAGGCTGTGGGTCGGAAC** |

*****[Zhang et al., 2008](#_ENREF_32).
